# Toxoplasmosis and knowledge: what do the Italian women know about?

**DOI:** 10.1017/S0950268820002393

**Published:** 2020-10-07

**Authors:** A. Martini, E. Pietrafesa, B.M. Rondinone, S. Iavicoli, S. D'amelio, S. Cavallero, M. Bonafede

**Affiliations:** 1 Department of Occupational and Environmental Medicine, Epidemiology and Hygiene, National Institute for Insurance against Accidents at Work, Rome, Italy; 2 Department of Public Health and Infectious Diseases, Sapienza University of Rome, Rome, Italy

**Keywords:** E-research, knowledge, protozoan disease, *Toxoplasma gondii*, Toxoplasmosis, women

## Abstract

Toxoplasmosis is a worldwide zoonotic infectious disease caused by *Toxoplasma gondii*. This infection is estimated to affect about a third of the world's population. The aim of this study was to evaluate the knowledge of Italian women about toxoplasmosis and its forms of transmission, clinical manifestations, diagnosis and prevention through two different modalities (e-research and traditional research). In a cross-sectional study, 808 Italian women were interviewed, using a self-administered questionnaire, through two different modalities: an e-research or web survey and a traditional paper research and 84% reported to have heard about toxoplasmosis, but from most of the sample, it resulted that the knowledge of the protozoan disease was superficial and incomplete.

The assessment of the dimensionality related to the toxoplasmosis knowledge's instrument showed that the scale is composed by two stable and reliable factors which explain 58.6% of the variance: (a) the basic knowledge (*α* = 0.83), which explains the 45.2% of the variance and (b) the specialist knowledge (*α* = 0.71), which explains the 13.4% of the variance. The variance and the multiple linear regression data analysis showed significant predictors of correct basic knowledge of toxoplasmosis: the highest age, the highest degree of study, to have previously contracted illness or to know someone who had contracted it, to be working or to be housewives. In conclusion, this study showed limited awareness of toxoplasmosis and suggested the implementation of effective education and learning programs. The results also showed that online data collection, in academic research, might be a valid alternative to more traditional (paper-and-pencil) surveys.

## Introduction

Toxoplasmosis is a worldwide zoonotic infectious disease caused by *Toxoplasma gondii (T. gondii*), an obligate intracellular parasite. It is estimated that about one-third of the world's population is infected with *T. gondii* and seroprevalence estimates a range from less than 10% to over 90% [1]. The seroprevalence of *T. gondii* varies according to age, geographical area, climate, culture, eating habits, animal husbandry and socioeconomic status of a certain population [2, 3].

According to the last European Centre for Disease Prevention and Control (ECDC) Annual Epidemiological Report (2016) related to the congenital toxoplasmosis data (ECDC), 242 confirmed cases of congenital toxoplasmosis were reported in the EU/EEA, with France amounting for the 81% of all confirmed cases, due to the active screening of the pregnant women. The notification rate was 6.7 cases per 100 000 live births, with the highest rates in France (24.9) followed by Poland (5.2). In the European Union (EU) 5148 million babies were born in 2016 and this surveillance report was based on congenital toxoplasmosis surveillance data collected in 2017 by the European Food and Waterborne and Zoonoses (FWD) Network. Twenty-two EU/EEA Member States reported congenital toxoplasmosis data to TESSy (21 EU Member States plus Iceland). In Denmark, Italy, the Netherlands, Norway, Portugal and Sweden a nationwide epidemiological surveillance system for congenital toxoplasmosis is still lacking [4].

In Italy, the surveillance system for congenital toxoplasmosis reporting data on live new-borns from mothers with gestational toxoplasmosis is active only from regional basis (i.e. Campania region) [5, 6] and the seropositivity rates range from less than 40% to over 80% and increase with age in women of the childbearing age range, representing a continuity of risk exposure.

However, a trend toward a decrease has become evident about *T. gondii* prevalence in women of childbearing age range since 2001 [7]. This reduction in prevalence is correlated to the declining incidence of the disease, due to lower exposure to the parasite changing in nutritional habits and improving hygiene practices. In fact, the *T. gondii* can be transmitted to humans through three main ways: (a) the ingestion of tissue cysts in undercooked meat of intermediate hosts [8, 9]; (b) the ingestion of the sporulated oocysts via soil, water, or food contaminated with faeces of infected and shedding cats and other felines (the definitive host) [8]; (c) the vertical transmission which causes congenital toxoplasmosis. It can result in foetal death and abortion, stillbirth or result in major ocular and neurological sequelae, ranging from slightly diminished vision to more severe disorders, such as retinochoroiditis, hydrocephalus and intracerebral calcifications [10–12].

Most of the *T. gondii* infections in immunocompetent individuals of most host species including humans are subclinical, thus healthy persons do not develop the clinically evident disease. On the contrary, the infection can lead to severe clinical manifestations and mortality, especially in the immunocompromised and in children with congenital infection [11].

Torgerson *et al*. showed that the global estimated incidence of congenital toxoplasmosis was about 190 100 annual cases (95% credible interval, CI 179 300–206 300). From the annual incidence, the global burden was calculated to be 1.20 million DALYs (95% CI 0.76–1.90) per annum. [13].

There is no commercial vaccine against toxoplasmosis, so the adoption of preventive measures is highly important: (a) the primary prevention aimed through the adoption of hygiene and food measures, in particular, during pregnancy; (b) the secondary prevention applied with the serological screening of pregnant women in order to identify early and treat the disease; (c) the tertiary prevention through the diagnosis, therapy and follow-up of the new-born. Because the disease is usually asymptomatic, the education and the prevention of pregnant women are critical in order to reduce infection rates [14].

The aim of this study was to evaluate the knowledge of Italian women about toxoplasmosis and its forms of transmission, clinical manifestations, diagnosis and prevention through two different modalities (e-research and traditional research) following an analysis of the state of the art on the topic.

## Materials and methods

### Literature search

From January 2010 to June 2020, the English and Italian articles about toxoplasmosis issues, indexed in PubMed, Google Scholar, Science Direct, Scopus, Medline were collected.

The Keywords were chosen, based on MeSH terms and were categorised in three groups as follows: ‘Education’ OR ‘Knowledge’ AND ‘Toxoplasma’ OR ‘Toxoplasmosis’.

Selected papers were carefully reviewed and the information included the first author, the year of publication, the location of study, the language and the sample size have been extracted.

### Study design and study population

A cross-sectional study was conducted (period 15 September–15 October 2018) through two different modalities: (a) an E-Research (ER) or web survey, a research conducted through social networks (Facebook, Instagram, Twitter and WhatsApp) and (b) a traditional research or in-person survey conducted through Paper Investigation (PI).

A self-administered questionnaire was used for the survey's data collection. The participants in the survey were women aged from 16 to 50 years old who could understand the questionnaire and fill in their answers without assistance.

Overall, 808 participants were recruited to complete and to return the survey questionnaire.

### Questionnaire

A structured Italian questionnaire was constructed and used for the data collection. In order to elaborate on this investigation tool, some national and international questionnaires, used in previous studies with the same objective, were selected and chosen [14–20].

In particular, from the experience of two studies conducted previously [21, 22], a questionnaire *ad hoc* was developed. A pre-testing conducted on a random sample allowed the optimisation of the instrument and to determine the time needed to complete the questionnaire too. The latest version of the questionnaire consisted of 27 closed-ended questions divided into two sections (general information; knowledge of parasite and parasitic disease) which aimed to collect from the respondents the following information:

1. ‘general information’ including social network contact modes; age; gender; education; date and place of birth; district of permanent residence; occupational and employment status; nationality; living in an urban or rural environment; contact with cats; subjects with previous *Toxoplasma gondii* infection or friends/relations with the parasitic disease (13 questions).
2. ‘knowledge of parasite and parasitic disease’ including to have heard or seen information about toxoplasmosis and through which kind of information resources; knowledge about the parasite, its life cycle and transmission routes; animal involved in the toxoplasmosis transmission; general clinical; diagnostic and prevention aspects of toxoplasmosis (14 questions).

### Questionnaire administration

For the online questionnaire administration, a Google account was created using the dedicated application (Drive) choosing the option for Google Modules and created the file named ‘Toxoplasmosis: assessment of knowledge’. The questionnaire was divided into two sections and questions were included according to the structure, type and mode of response. After a control phase, the questionnaire thus created was placed online and generated a link for the subsequent web dissemination (shipment via mail or through the social media platforms). At the same time, a Facebook page (https://www.facebook.com/toxoplasmosi) dedicated to the research survey was created and shared over a number of networks contacts to invite respondents to the online auto compilation through these social media platforms: Instagram, WhatsApp, Twitter, E-mail due to the specific target of the survey.

In the first page of the questionnaire an introductory section within the information about the project, the objectives and target of the study, as well as the methods of data management that would ensure full compliance with national and European legislation on privacy (GDPR-application from 25 May 2018) was included.

At the end of the survey, in order to perform more detailed statistical analysis from Google Modules, an Excel file with all transferred responses was retrieved.

The paper administration of the same investigative tool during the same period involved a sample of university students. After compiling and withdrawing at the time, all the acquired data of the paper questionnaires were entered manually on spreadsheets Excel was prepared with the same structure as the online data file. The questionnaire tooks approximately 15 minutes to be completed. The survey questionnaire was preliminary tested in early September 2018 (*n* = 50) to ensure the practicability, the comprehension and the interpretability of answers. The questionnaire was slightly refined for wording and format before distribution to the study sample based on the results of the pre-testing, in consideration that the items were translated into Italian.

### Statistical analysis

The socio-demographic variables (age categories, education, nationalities, macro geographical area, living environment and employment status) were analysed separately in the two samples the e-research/web survey (Group 1) and the traditional research (Group 2), applying the Chi-Square test to check the presence of an association between the variables themselves.

As the two sample groups (Group 1 and Group 2) are very different from almost all the socio-demographic variables analysed, the analyses were conducted separately.

Relating to the knowledge of the parasite and of the parasitic disease, the responses to the items were re-coded in ‘correct’, ‘partially correct’ and ‘incorrect’ knowledge. Descriptive analyses and normality analyses of the items in this section were conducted. To assess the dimensionality of the knowledge instrument, the principal component analysis with Varimax rotation was carried out and the Cronbach's Alpha calculation allowed an empirical assessment of the reliability. Through the analysis of Student's *t* test and variance (One-Way ANOVA), carried out separately by the method of administration, the possible differences with socio-demographic variables were examined. The multiple linear regression model was performed to explore the significant predictors associated with the main knowledge factors by weighing the models for the mode of administration questionnaire. Significant values of *P* < 0.05 were considered.

## Results

### Literature research

From January 2010 to June 2020, 43 articles about toxoplasmosis issue were found and reviewed. Table 1 shows the outcomes of the literature research.

**Table 1.** Literature research findings (2010–2020)

Authors	Publication year	Place	Target	Sample size
Ziemba *et al*. [23]	2010	Łódź, Polonia	Pregnant women, midwives, medical students and health professionals (obstetricians)	310
Dabritz *et al*. [15]	2010	California, USA	General population	54
Thaller *et al*. [24]	2011	Rome, Italy	Pregnant women	2356
da Silva *et al*. [16]	2011	Rio de Janeiro, Brazil	Health professionals (physicians and nurses)	112
Pereboom *et al*. [25]	2013	Netherlands	Pregnant women	1097
Ogendi *et al*. [17]	2013	Thika District, Kenya	Farmers households	385
Amin *et al*. [26]	2013	Al Hassa, Saudi Arabia	Pregnant women	872
Bresciani *et al*. [27]	2013	São Paulo, Brazil	Teachers (from elementary schools)	30
Millar *et al*. [28]	2014	Rio de Janeiro, Brazil	Pregnant and postpartum women	400
Velázquez-Hernández *et al*. [29]	2014	Sancti Spiritus, Cuba	Women of childbearing age and pregnant women	119
Contiero-Toninato *et al*. [14]	2014	Paraná, Brazil	Health professionals (physicians and nurses) and pregnant women	410
Andiappan *et al*. [30]	2014	Malaysia, Philippines, and Thailand	Pregnant women	2598
Elsafi *et al*. [31]	2015	Drahran, Saudi Arabia	Pregnant women	400
Ebrahimi *et al*. [32]	2015	Mashhad, Iran	Female students	549
Hung *et al*. [33]	2015	Taipei, Taiwan	Pregnant women	104
Al Rashada *et al*. [34]	2016	Al Ahassa, Saudi Arabia	Female students	88
Ansari Lari *et al*.[35]	2016	Shiraz, Iran	Female students	200
de Moura *et al*. [36]	2016	Rio de Janeiro, Brazil	Pregnant women	405
Lehmann *et al*. [37]	2016	Rio Grande do Sul state, Brazil	Pregnant and postpartum women	100
Yun *et al*. [38]	2016	Wixi, Jiangsu, China	Pregnant women	217
Al-Sheyab *et al*. [39]	2015	Jordan	Female students	1390
Yan-Li *et al*. [40]	2017	Changzhou, Jiangsu, China	Pregnant women and patients with neoplasia	300
de Moura *et al*. [21]	2017	Niterói, Rio de Janeiro, Brazil	Health professionals and pregnant women	641
Mayada *et al*. [41]	2017	Tikrit, Iraq	Females	200
Swaileh *et al*. [19]	2017	Gaza Strip, Palestine	Female students	976
Alvarado-Esquivel *et al*. [18]	2017	Durando, Mexico	Clinical laboratory professionals	192
Efunshile *et al*. [42]	2017	Ogun, Ebonyi, Anambra, Nigeria	Health professionals (medical doctors)	522
Sousa *et al*. [43]	2017	São Luís, Maranhão, Brazil	Pregnant women and health professionals (nurses)	30
Avelar *et al*. [44]	2018	Goiás, Brazil	Postpartum women	229
El-dessouki *et al*. [45]	2018	Minya, Egypt	Pregnant women	200
Smereka *et al*. [46]	2018	Wroclaw, Poland	Pregnant women	465
Dairo *et al*. [47]	2018	Ibadan, Nigeria	Pregnant women	377
Yam L, *et al*. [22]	2018	Kuala Lumpur, Malaysia	Urban and rural population	321
Mahfouz *et al*. [20]	2018	Jazan, Saudi Arabia	Female students	440
Moura *et al*. [48]	2019	Maranhão, Brazil	Pregnant women	239
Velázquez-Hernández *et al*. [28]	2019	Durango City, Mexico	Housewives	185
Onduru *et al*. [49]	2019	Dar Es Salaam, Tanzania	Pregnant women and health professionals	393
Al-Hellaly *et al*. [50]	2019	Babil, Iraq	Pregnant women	98
Wasik M [51]	2019	Kielce, Poland	Woman	63
Ouzennou *et al*. [52]	2019	Marrakech, Morocco	Pregnant women	16
Laboudi *et al*. [53]	2020	Rabat, Morocco	Health professionals (medical doctors, nurses, midwives and laboratory technicians)	96
Senosy [54]	2020	Beni Suef, Egypt	Female students	1079
MarwaAbdElmonaem *et al*. [55]	2020	Menoufia, Egypt	Health professionals (nurses)	88

### The present study

The survey sample of our study consisted of 808 female subjects, divided into two groups, according to the method of questionnaire administration: 604 (74.8%) completed the online questionnaire (Group 1) and 204 (25.2%) the traditional paper version (Group 2). Table 2 shows the sample description. For the online survey dissemination, different multimedia channels and social platforms were used: Facebook (76.7%), Whatsapp (12.3%), E-mail (8.3%), Instagram (2.6%) and other channels (0.2%).

**Table 2.** Sample description

	Group 1 Online administration (*N* = 604) n (%)	Group 2 Paper and pencil administration (*N* = 204) *n* (%)	*P*-value χ^2^ test
Age categories			
<24 yrs	122 (20.2%)	174 (85.3%)	***P* < 0.001**
25–34 yrs	244 (40.4%)	29 (14.2%)	
>35 yrs	238 (39.4%)	1 (0.5%)	
Mean age (SD)	32.3 (7.9)	21.6 (2.9)	
Education			
Middle/high school diploma	259 (42.9%)	159 (78.3%)	***P* < 0.001**
Degree and post graduate	345 (57.1%)	44 (21.7%)	
Nationality			
Italian	585 (96.9%)	197 (96.6%)	*P* = 0.842
Foreign	19 (3.1%)	7 (3.4%)	
Macro geographical area			
Northern Italy	128 (21.5%)	5 (2.5%)	***P* < 0.001**
Central Italy	224 (37.6%)	159 (79.9%)	
Southern Italy	244 (40.9%)	35 (17.6%)	
Living environment			
Urban	491 (81.3%)	188 (92.6%)	***P* < 0.001**
Rural	113 (18.7%)	15 (7.4%)	
Employment status			
Student	120 (20.1%)	204 (100.0%)	***P* < 0.001**
Worker	386 (64.5%)	–	
Housewife	44 (7.4%)	–	
Unemployed	48 (8.0%)	–	
Previous *Toxoplasma gondii* infection or friends/relations with disease			
Yes	179 (29.8%)	21 (10.6%)	***P* < 0.001**
No	422 (70.2%)	178 (89.4%)	
Having heard about toxoplasmosis			
Yes	579 (95.9%)	105 (51.7%)	***P* < 0.001**
No	25 (4.1%)	98 (48.3%)	

From the application of the Chi-Square test, it has emerged that the subjects belonging to the two groups were composed in a different way with respect to all socio-demographic variables (*P* < 0.001) excluding the nationality (*P* = 0.842).

The highest age categories (85.3%) were made up of subjects under the age of 24 in Group 2 while in Group 1, the 40.4% of the subjects were between 25 and 34 years old; the 39.4% of the respondents was over 35 years old while the 20.2% was under 24 years old.

In addition, in Group 1, approximately 43% had a middle school or high school diploma and 57.1% had a bachelor's, master's or post-graduate degree. In Group 2, these percentages were 78.3% and 21.7%, respectively.

With regard to the macro geographical area, in Group 1, 40.9% of the subjects came from the Southern Italy and Islands, 37.6% from the Central Italy and 21.5% from the Northern Italy. Conversely, in Group 2 the main share (about 80%) came from the Central Italy.

The type of environment in which respondents lived predominantly was urban in both groups: 81.6% in Group 1 and 92.6% in Group 2. The variable that characterised the two subgroups in a clear way was represented by the work carried out; Group 2 was made up entirely of students, while in Group 1, 64.5% were workers and 20.0% were student. Considering the subjects who had already personally contracted the disease or who knew someone who had contracted it, the percentage frequencies were around 30% in Group 1 and 11% in Group 2. The most commonly used sources of knowledge in the online sample were: medical specialist (48.8%); books (32.6%); internet (26.5%); general practitioner (14.6%); television (14.4%); newspapers (10.1%); veterinarian (10.1%); other healthcare professional (8.1%) and radio (1.0%). The information resources in the paper sample were: television (20.6%); internet (16.7%); books (12.7%); newspapers (5.9%); general practitioner (4.9%); veterinarian (4.9%); medical specialist (3.4%); radio (2.0%) and other healthcare professional (1.5%).

As the secondary analysis showed, further significant predictors of a correct general knowledge of toxoplasmosis were to have previously contracted the disease or to know someone who contracted it. That was, a further source of knowledge, in accordance with previous studies, were acquaintances and family members.

From the analysis of the main components carried out (see Table 3) the knowledge scale was composed of two stable and reliable factors that explained 58.6% of the variance:

- the first factor (*α* = 0.83), which explained the 45.2% of the variance, was called ‘General knowledge’ because it brought together all the items concerning the most common basic knowledge of the protozoan disease and summarised the information relating to the diagnosis, the animal involved in the transmission, how to contract the disease, how to prevent it and the period of pregnancy;
- the second factor (*α* = 0.71), which explained the 13.4% of the variance, was called ‘Specialist knowledge’ because it brought together all the items that concerned more in-depth and less widespread knowledge of the disease, and summarised information relating to the effects on the foetus, the effects on the woman and the symptoms.

Analysis of the Student's *t* test and variance (One-Way ANOVA), conducted separately by mode of administration (See Table 4), revealed significant differences in the knowledge factors relative to the study; the age categories; the working conditions and the fact that subjects have contracted or know someone who had contracted toxoplasmosis. In particular, it is noted that both types of knowledge were more correct among women with a degree or post qualification in the ER sample. There is a more correct general knowledge in both samples among women with the highest age, whereas the two types of knowledge were more correct among women workers and housewives and among those who have contracted or know someone who had contracted the disease.

**Table 3.** Main component analysis of the scale ‘Knowledge’

	Component	
	1 - ‘General knowledge’	2 - ‘Specialist knowledge’
Diagnosis (How is toxoplasmosis diagnosed?)	0.770	
Animal (Which animal is involved in the transmission of toxoplasmosis?)	0.752	
In what period (Toxoplasmosis is a disease with more severe consequences if…is contracted)	0.751	
How to contract the disease (How can the human being contract the disease?)	0.744	
Way to prevent (How can it be prevented?)	0.715	
Pregnancy period (What is the period of pregnancy in which the disease can cause more complications if contracted?)	0.555	
Female effects (What may be the effects/symptoms on the woman?)		0.884
Foetal effects (What may be the effects on the unborn child?)		0.746
Symptoms (What are the symptoms associated with the disease?)		0.677
Extraction method: Main component analysis. Rotation method: Varimax with Kaiser normalisation.

**Table 4.** Student's *t* test and variance analysis (one-way ANOVA) between knowledge factors and socio-demographic variables

Variables	General knowledge (1 = corrected; 2 = partially corrected 3 = incorrect)		Specialist knowledge (1 = correct; 2 = partially correct 3 = wrong)	
Mean	SD	Mean	SD
Have you contracted or know someone who contracted the disease?	Group 1	Yes	1.38	0.30	2.24	0.45
		No	1.53	0.47	2.37	0.49
	test; *P*-value		*t* = −4.16; *P* < **0.001**		*t* = −3.08; *P* < **0.005**	
	Group 2	Yes	1.90	0.45	2.57	0.37
		No	2.42	0.57	2.83	0.27
	test; *P*-value		*t* = −4.10; *P* < **0.001**		*t* = −3.31; *P* < **0.001**	
Education	Group 1	Middle/high school diploma	1,55	0.48	2.40	0.44
		Degree and post graduate	1.44	0.39	2.29	0.50
	test; *P*-value		*t* = 3.13; *P* < **0.005**		*t* = 2.70; *P* < **0.05**	
	Group 2	Middle/high school diploma	2.41	0.59	2.82	0.29
		Degree and post graduate	2.25	0.57	2.73	0.30
	test; *P*-value		*t* = 1.59; *P* = 0.11		*t* = 1.64; *P* = 0.10	
Age categories	Group 1	<24 yrs	1.81	0.56	2.32	0.47
		25-34 yrs	1.41	0.38	2.32	0.47
		>34 yrs	1.40	0.33	2.36	0.49
	test; *P*-value		*F* = 49.69; *P* < **0.001**		*F* = −0.56; *P* = 0.57	
	Group 2	<24 yrs	2.41	0.58	2.79	0.31
		25-34 yrs	2.21	0.51	2.84	0.21
		>34 yrs	–	–	–	–
	test; *P*-value		*F* = 3.27; *P* < **0.05**		*F* = 1.52; *P* = 0.22	
Employment status	Group 1 + Group 2	Student	2.16	0.64	2.60	0.46
		Worker	1.41	0.36	2.33	0.49
		Housewife	1.33.	0.32	2.42	0.39
		Unemployed	1.49	0.39	2.50	0.42
	test; *P*-value		*F* = 125.15; *P* < **0.001**		*F* = 20.46; ***P* = 0.001**	

Multiple linear regression analysis showed significant results only for the ‘General knowledge’ factor (*R*^2^ = 0.79). The model confirmed the previous highlights: the highest age; the highest degree of study; to have previously contracted illness or to know someone who had contracted it; to be working or to be housewives, were significant predictors of correct ‘General knowledge’ of toxoplasmosis (see Table 5).

**Table 5.** Linear regression model: ‘General knowledge’ as a dependent variable

Model – Dependent variable: ‘General knowledge’	Beta	*t-value*	*P*-value
(Steady)		2.332	0.020
Age	−0.115	−3.193	0.001
Education	−0.064	−2.291	0.022
Previous toxoplasma infection or friends/relations with parasitic disease	0.868	46.846	0.000
Employment status	−0.331	−12.369	0.000

*F* = 766.731; *P* < 0.001; *R*^2^ = 79%; adjusted *R*^2^ = 79%.

## Discussion

The analysis of the national and international literature showed that there were not many studies conducted on the knowledge of the parasite and the disease caused by *Toxoplasma gondii* and many of these that were dated, carried out only on specific targets (i.e. pregnant women, healthcare personnel, students, housewives, etc.) and with samples that were not particularly numerous.

To the best of our knowledge, this was the first study conducted through an online survey (e-research) about the knowledge of toxoplasmosis and addressed to women in the general population.

The e-research is a new investigative tool, widely used in Countries with high internet usage. Also according to the literature data, the advantages of e-research over a traditional study (telephone, post or personal interview) are: (a) speed of detection (the online survey times are certainly lower than research carried out in a traditional way); (b) monitoring and real-time analysis of the data (following the insertion/recording of the data, a summary and immediate analysis of the trend is possible); (c) cost-effectiveness (internet interviews are cheaper than similar surveys conducted using traditional methods); (d) reduction of intrusiveness of detection (an online questionnaire is a tool to which the user has decided to answer behind the prompt of very few external agents; this improves the fidelity and spontaneity of the answers); (e) achievement of specific targets favouring the communicative specificity of the survey; (f) use of multimedia (sound, pictures and movies) [56, 57]. In fact, as this study highlighted, during the same time frame (1 month) a quarter of the subjects were reached through a traditional paper compilation and three quarters through the online survey; the online data collection has enabled preliminary analyses to be carried out to verify and monitor the results and any difficulties in entering/recording the data; the sample reached through the paper administration was very young, narrow, very homogeneous and less representative (e.g. limited age categories, no workers presents, same schooling attendance, etc.).

This study showed that about 84% of women interviewed, reported to hear about toxoplasmosis, while a small percentage of about 15.3% of the sample reported not to know the toxoplasmosis. This condition places these women in a high-risk group, as the complete lack of information could allow exposure to the parasite because of a possible pregnancy. This figure was in line with the most recent results obtained in different studies conducted in Europe. In fact, a study conducted in 2015 in Geneva (Switzerland) showed a high knowledge of toxoplasmosis among pregnant women (87%) included in the study [58]. Another study conducted in Poland in 2018 even reported a higher basic knowledge of toxoplasmosis among pregnant women (94.4%) [46].

Although a large number of women reported having heard about toxoplasmosis, knowledge of protozoan disease resulted in most of the sample to be incomplete and superficial. This data was evident for the ‘General knowledge’ (diagnosis, type of animal involved, mode of transmission of the parasite, prevention of infection and pregnancy period) but it was even more so in relation to the ‘Specialist knowledge’ (signs and symptoms of the disease, effects on the foetus and effects on the woman). This aspect was important because although toxoplasmosis in pregnancy is predominantly asymptomatic, the recognition, in particular of early signs and symptoms (e.g. fever, asthenia, headache, muscle pain, weakness, lymphadenopathy and other flu-like symptoms, rarely visual changes) may lead to an early diagnosis of the disease and thus reduce the risk of transmission of infection from the mother to the child. Furthermore, to know the effects on the foetus and on the woman allows to improve the perception of risk and to translate knowledge into preventive behaviours.

The inadequate knowledge of toxoplasmosis found in this study was consistent with the results of previous studies.

In the study of Millar *et al*., although 72.2% of women claimed to know toxoplasmosis, more than 80% of women participants did not know the symptoms of the disease and less than half reported to know how to transmit the parasite [28]. In another study conducted in 2017 in Iraq, most of the respondents did not know the parasitic disease accurately nor they were sure of the signs and symptoms that it can cause in the mother and child [41]. In addition, a very recent study, conducted by Mahfouz *et al*., showed that a substantial part of the students of Jazan University was not aware of the symptoms of toxoplasmosis [20]. The latter result was the same in other studies conducted in the USA, Malaysia, the Philippines and Thailand, Iran and Saudi Arabia too [30–32, 34, 59].

Some of the significant predictors of a correct ‘General knowledge’ of toxoplasmosis emerged from the analysis of the data of the present study, were the age and the title of the study. In accordance with these findings, Smereka *et al*. showed in a study conducted in Poland that among pregnant women the youngest age, the residence in the city, the level of higher education and the number of children were significantly associated with a better understanding of the symptoms of toxoplasmosis [46]. A previous study conducted in Brazil on 405 pregnant women, aimed at knowing the factors associated with better knowledge of toxoplasmosis, showed that the percentage of pregnant women reporting better knowledge of protozoan disease increased with the age, the education level and the pregnancy number [36]. The same results were observed, in a study conducted in 2017 by the same author, on 500 pregnant women in Niteroi, State of Rio de Janeiro [21]. Recent research, conducted on 440 female students in Saudi Arabia by Mahfouz *et al*. highlighted that in accordance with previous literature studies, knowledge of toxoplasmosis increases with age [20, 59, 60] and with a high educational stage [20, 60].

The level of education, as described in the literature, influences not only the knowledge but also the seroprevalence estimates to *T. gondii*: pregnant women with a lower level of education have higher seroprevalence estimate than those with higher schooling. Consequently, pregnant women with a higher educational level appear to have a more accurate knowledge of protozoan disease in order to prevent it [33].

It also emerged from our study that the working environment was a significant predictive variable for ‘General knowledge’. In particular, the results showed a more correct ‘General knowledge’ among housewives than female workers, students and unemployed women. This result highlights the need to implement all information/training and risk management actions to protect the health of women workers, especially because of physiological conditions (pregnant worker) or pathological (immunocompromised workers). In particular, as several studies highlighted, in specific occupational sectors (such as farmers, butchers, veterinarians, animal handlers, zoo attendants, slaughterhouse workers, meat inspectors, cooks and others contacting raw meat, grounds, landscapers and gardeners, laboratory workers, waste pickers and waste workers, etc.), workers are potentially exposed to the risk of *T. gondii* and therefore to develop the disease [61–67].

The occupational assessment of risk factors, in particular for biological risk, is necessary for the first step of the risk prevention and management. In this context policies and procedures should be periodically reviewed (e.g. employing measures of personal hygiene) to minimise and to prevent zoonotic transmission.

To the best of our knowledge, according to our survey, only a few studies were conducted to evaluate the knowledge about toxoplasmosis in the workplace potentially exposed to the risk; further studies need to be implemented.

Thus, in general, the knowledge of the socio-demographic profile of a population of women of childbearing age range is very important for the planning and implementation of primary and secondary prevention actions to counter the spread of infestation by *T. gondii* [44, 68].

Our research also analysed the sources of knowledge of *T. gondii* and toxoplasmosis in the two samples under investigation. The most commonly used sources for knowledge were in the online sample doctors books and internet, while in the paper sample were television, internet and books. As the secondary analysis highlighted, further significant predictors of a correct ‘General knowledge’ of toxoplasmosis were to have previously contracted the disease or to know someone who had contracted it. That was, a further source of knowledge, in accordance with previous studies, were acquaintances and family members. In fact, Jones *et al*. showed that 53% of participants in the study, conducted in the USA, reported receiving information from a doctor and 45% from family/friends [59]. While Ogunmodede *et al*. reported that 63% of the studied sample received information from a doctor and 35% from family/friends [60]. Following studies have shown that books, magazines and internet were also important sources of information for pregnant women in relation to toxoplasmosis [45] and that the health professionals played an important role in informing women about preventable infectious diseases such as the disease caused by *T. gondii* [41].

An important result of our findings was to highlight the relationship between the ‘General knowledge’ of toxoplasmosis and the area of residence (significant predictor). In particular, people living in urban areas have generally a higher knowledge of toxoplasmosis than people living in rural ones. This is important because the rural residents are more exposed to agricultural work and gardening and indicate that greater attention should be paid to the health education among the residents of the extra-urban areas, in order to allow them to avoid contracting the disease [50]. In fact, it is suspected, based on the literature, that the geo-demographic state has a direct impact on the seroprevalence estimates of the disease, because of the contact with the soil is more frequent in the rural areas. In two studies carried out in Colombia and China, toxoplasmosis was associated, in women of the childbearing age range, with the residence in the rural areas [69, 70]. Because of these findings, considering the number of women susceptible to *T. gondii* infection during pregnancy and who could vertically transmit the parasite to the child, the primary prevention is the desirable public health measure. In fact, if the risk knowledge is low (toxoplasmosis), the number of people who are aware of the risk factors associated with the infection is reduced, consequently the percentage of people who take hygiene and dietary measures, that could prevent the infection during the pregnancy or immunodepression, endangering one's own health and that of the foetus. Hence the need for health education programs.

Pawlowski *et al*. [71] and Carter *et al*. [72] have drawn attention to the importance of educational programs in the prevention of congenital toxoplasmosis among the women of reproductive age to reduce the seroprevalence of *T. gondii* [25, 26, 73, 74]. In line with these studies, Elsafi *et al*. showed that about 3/4 of the 400 pregnant Saudi women, participating in the study, had never heard of toxoplasmosis and presented a toxoplasmosis risk 4.04 times greater than those who knew about the protozoan disease [31]. Even pregnant women studied in Niterói, Rio de Janeiro, who reported knowledge of toxoplasmosis, showed a significantly lower probability of being seropositive to *T. gondii* [28]. A previous study in Belgium, on primary prevention of congenital toxoplasmosis, revealed 63% reduction in the rate of seroconversion during pregnancy after the use of activated hygienic measures following an intense educational program [74]. Further studies have highlighted the importance of health education among pregnant women aimed at reducing the seroprevalence of this disease and, consequently, to minimise the adverse effects of infection in the foetus or newborn [25, 26, 73].

Based on our results and literature data analysis, the effectiveness of an educational programme that involves changes in lifestyle depends on the widespread and repeated information on risk factors that need to be adapted to the socio-demographic traits and cognitive needs of the community to which they are directed for [75]. Indeed, the positive effect of educational materials on behavioural changes depends on the age, the education of the population and the individual motivation that leads to a better understanding of the disease and consequently to adopt a healthier lifestyle, especially during the pregnancy [71, 76].

In conclusion, the results of this study showed limited awareness of *T. gondii* in the population/in women and toxoplasmosis and suggested to promote effective education and learning programs aimed at the prevention of the disease ‘to know’ (to increase the knowledge) and ‘to do’ (to change the attitudes). The results also showed that online data collection, in academic research, might be a valid alternative to more traditional (paper-and-pencil) surveys.

## Limitations of the study

The main limitation of the study is the non-assessment of the participants' risk procedures, the practices and the habits. Another limit could be considered the time monitoring of the online compilation to verify if every single participant used additional sources of information to answer to the survey. However, this situation exposes to an overestimation of the knowledge and not to an underestimation of the same-one.

## Data Availability

The data that support the findings of this study are available from the authors upon request.

## Supplementary material

For supplementary material accompanying this paper visit http://dx.doi.org/10.1017/S0950268820002393. click here to view supplementary material
