# Supplementary material for: Toxoplasmosis and knowledge: what do the Italian women know about?
Source: Epidemiol Infect. 2020 Oct 7;148:e256. doi: 10.1017/S0950268820002393 (PMC7689595; doi:10.1017/S0950268820002393)
Supplement: Supplementary file 1 [file S0950268820002393sup001.docx]

**Appendix**

*Questionnaire used in this study*

**SECTION “GENERAL INFORMATION”**

1. **In which way have you been contacted?** (*)^[[1]](#footnote-1)^

*Mark just one oval*

- I received an E-MAIL
- Via FACEBOOK
- Via WHATSAPP
- or Via INSTAGRAM
- Other: _____________________________

1. **Gender** (*)^[[2]](#footnote-2)^

*Mark just one oval*

- F
- M

1. **Year of birth** (*)^[[3]](#footnote-3)^ _____________________________
2. **Nationality** (*)^[[4]](#footnote-4)^ _____________________________
3. **Place and province of birth** (*)^[[5]](#footnote-5)^ _____________________________
4. **Place and province of residence** (*)^[[6]](#footnote-6)^ _____________________________
5. **You live in a……. environment** (*)^[[7]](#footnote-7)^

*Mark just one of the ovals*

- Rural
- Urban

1. **Educational qualifications** (*)^[[8]](#footnote-8)^

*Mark just one oval*

- Secondary/high school diploma
- Bachelor’s degree
- Master’s degree or specialist degree
- Post lauream (school of specialization, PhD, Master, …)

1. **Employment activities** (*)^[[9]](#footnote-9)^

*Mark just one oval*

- Yes
- No

1. **If NO** (*)^[[10]](#footnote-10)^

*Mark just one oval*

- Student
- Other: _____________________________

1. **If YES at which sector your work activity belongs?**

*Mark just one oval*

- Agriculture, forestry, livestock farming
- Food sector
- Manufacturing
- Water supply; sewerage, waste management and sanitation activities
- Construction
- Trade
- Accommodation and catering activities
- Professional, scientific and technical activities
- Health and social assistance
- Other: _____________________________

1. **Specific task carried out** _____________________________
2. **Have you contracted or know someone who has contracted the disease?**

*Mark just one oval*

- Yes, I contracted it
- Yes, I know someone who contracted it
- No

**SECTION “KNOWLEDGE OF PARASITE AND PARASITIC DISEASE”**

1. **Have you ever heard of toxoplasmosis before?**

*Mark just one oval*

- Yes
- No

1. **If YES, where?**

*Select all applicable entries*

- Books
- Magazines
- Television
- Radio
- Internet
- General practitioner
- Medical specialist (e.g. gynaecologist)
- Other healthcare
- Veterinarian
- Other: _____________________________

1. **Which animal is involved in the transmission of toxoplasmosis?**

*Select all applicable entries*

- Dog
- Cat
- Canary
- Hamster
- Mosquito
- Fish
- I don’t know

1. **Can the human being become infected?**

*Mark just one oval*

- Yes
- No
- I don’t know

1. **How can the human being contract the disease?**

*Select all applicable entries*

- Eating undercooked meat
- Eating unwashed vegetables
- Through the puncture with infected needle
- Through the tick bite
- Through coughing/sneezing
- I don’t know

1. **What are the symptoms associated with the toxoplasmosis?**

*Select all applicable entries*

- Headache
- Joint pain
- Vomiting
- Diarrhea
- Fever
- Rash
- I don’t know

1. **Toxoplasmosis is a disease with more serious consequences if it is contracted being…**

*Select all applicable entries*

- Pregnant
- Breastfeeding
- In old age
- In paediatric age
- I don’t know

1. **Do you think that the disease can be transmitted from the mother to the unborn child during pregnancy?**

*Mark just one oval*

- Yes
- No
- I don’t know

1. **If YES, what may be the effects on the unborn child?**

*Select all applicable entries*

- Hydrocephalus
- Retardation
- Calcification of the brain
- Chororetinite
- Miscarriage
- I don’t know

1. **If YES, what may be the effects/symptoms on the woman?**

*Select all applicable entries*

- Enlarged liver
- Enlarged spleen
- Enlargement of the lymph nodes
- Headache
- Muscle/joint pain
- I don’t know

1. **What is the pregnancy period in which the disease can cause more complications if contracted?**

*Select all applicable entries*

- First quarter
- Second quarter
- Third quarter
- Indifferent
- I don’t know

1. **How is toxoplasmosis diagnosed?**

*Select all applicable entries*

- Chest X-ray
- Blood draw
- Urinalysis
- Oropharyngeal swab
- Analysis of the symptoms
- I don’t know

1. **Is it possible to prevent toxoplasmosis?**

*Mark just one oval*

- Yes
- No
- I don’t know

1. **If YES how can it be prevented?**

*Select all applicable entries*

- To clean cat litter at least once a day
- To clean cat litter at least once a day
- To use gloves during gardening/horticulture
- To wash and peel fruits and vegetables before consumption
- To cook the meat well before eating it

1. Answer is required [↑](#footnote-ref-1)
2. Answer is required [↑](#footnote-ref-2)
3. Answer is required [↑](#footnote-ref-3)
4. Answer is required [↑](#footnote-ref-4)
5. Answer is required [↑](#footnote-ref-5)
6. Answer is required [↑](#footnote-ref-6)
7. Answer is required [↑](#footnote-ref-7)
8. Answer is required [↑](#footnote-ref-8)
9. Answer is required [↑](#footnote-ref-9)
10. Answer is required [↑](#footnote-ref-10)
